# Sturge Weber syndrome, when brain CT is enough for diagnosis: about a case

**DOI:** 10.11604/pamj.2020.36.308.24989

**Published:** 2020-08-20

**Authors:** Ibrahima Niang, Khadim Mbacké Ndiaye, Alassane Mamadou Diop, Ibrahima Faye, Mbaye Thiam, Coumba Laobé Ndao, Sokhna Ba

**Affiliations:** 1 Radiology Department, National University Hospital Center Fann, Dakar, Senegal,; 2 Neurology Department, National University Hospital Center Fann, Dakar, Senegal

**Keywords:** Sturge Weber, seizures, pial angioma, gyriform calcifications

## Abstract

One of the main manifestations of Sturge Weber syndrome is seizures. We report the case of a child received in the context of generalized seizures and in whom a cerebral contrast CT was sufficient to make the diagnosis of Sturge Weber syndrome.

## Introduction

Sturge-Weber disease is a neurocutaneous and ocular phacomatosis, with vascular malformation substratum. It is a rare disease (1 in 20,000 to 50,000 viable births), non-familial, congenital [1]. It combines in its complete form, a congenital planar angioma, a leptomeningeal homolateral capillarovenous angioma and a choroidal angioma. Neurological signs include 75 to 90% epilepsy, often early and severe [1, 2]. Brain imaging by section is essential and in particular MRI which is the benchmark examination in early detection as well as follow-up [3].

## Patient and observation

**Figure 1:**
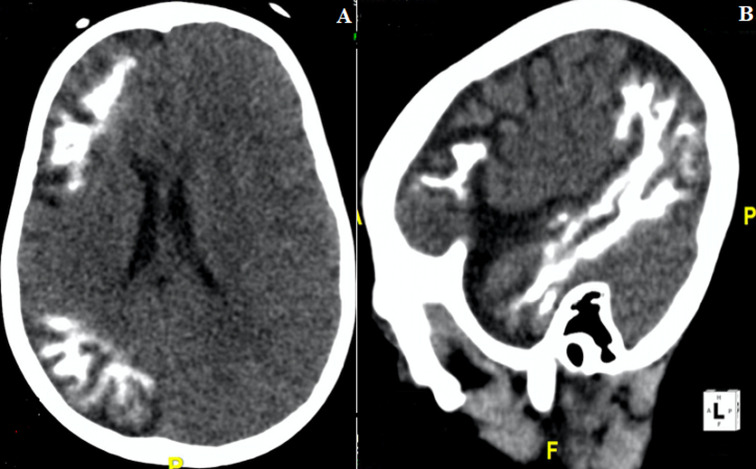
non contrast brain CT A) axial section showing a right hemi-cerebellar atrophy associated with gyriform fronto-parietal calcifications; B) right sagittal reconstruction showing these calcifications at the temporal lobe also

**Figure 2:**
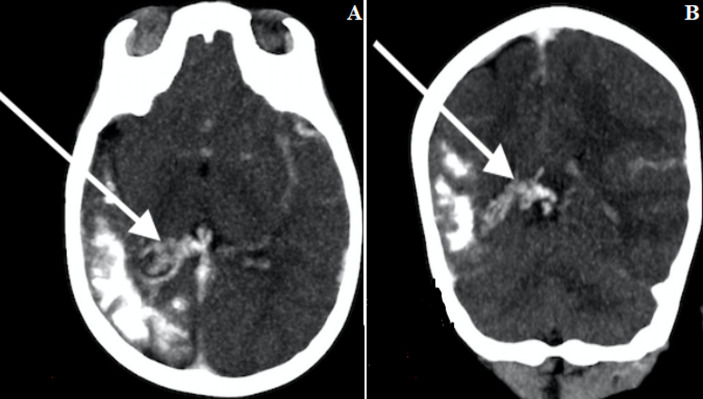
cerebral contrast CT in MIP A) (axial); B) (coronal) showing the right pial angioma in the form of an enlargement and dilation of the choroid vessels (white arrows)

We report the case of a boy aged 03 years and 05 months, referred to the imaging department for intense generalized non-febrile seizures, drug-resistant and evolving gradually since his first year of life. The patient was also mentally retarded and the examination found no cutaneous angioma or eye problems. No history of familial epilepsy or parental consanguinity was found. A contrast brain CT was carried out. It made it possible to highlight an atrophy of the right cerebral hemisphere, seat of multiple gyriform cortical calcifications predominantly frontotemporal (Figure 1). On the phases after injection of contrast, a hypertrophy and hyper vascularization of the right choroid plexus, corresponding to the choroidal angioma, is visualized (Figure 2). The importance of cerebral calcifications did not allow to highlight a possible pial angioma. No other brain or eye abnormalities were identified. In total, the gyriform cerebral calcifications associated with choroid angioma in this context of seizures in this child made it possible to make the diagnosis of Sturge Weber Syndrome.

## Discussion

Sturge-Weber syndrome (SWS), a rare congenital neurocutaneous syndrome, was first described in 1879 by Sturge in a patient with facial angioma, glaucoma and seizures [4]. The current definition of SWS includes a cutaneous angioma of the face reaching territory V1, neurological malformations (most often, ipsilateral leptomeningeal angioma, which can be responsible for convulsions, mental retardation or neurological deficit) and ophthalmological anomalies (angioma choroid, congenital glaucoma) of inconsistent presence [5, 6]. Roach *et al*. [7] distinguished 3 types of Sturge Weber disease: type 1 (classic, complete form): neuro-oculo-cutaneous involvement; type 2 (bi-symptomatic form): facial angioma and ophthalmic involvement without leptomeningeal angioma; type 3 (rough form): pial angioma without facial or ophthalmic involvement. The clinical form presented by our patient corresponded to type 3 with exclusively neurological signs without cutaneous angioma or ocular abnormality. On cerebral computed tomography, the cerebral atrophy associated with gyriform hemispherical calcifications were typical enough to confirm the diagnosis despite the impossibility of visualizing the pial angioma through these calcifications. Choroidal angioma, on the other hand, is well visualized by cerebral CT with injection of contrast product. This illustrates that in a situation of unavailability of magnetic resonance imaging, brain CT can be a good compromise for the exploration of Sturge Weber disease.

## Conclusion

This case shows that even if MRI remains the examination of choice in the radiological exploration of Sturge Weber syndrome, cerebral CT if it is sufficiently characteristic can be enough to confirm this diagnosis and thus be a compromise where the MRI is unavailable.
